# Eine Frage des Kindeswohls: Wie die Corona-Zeit und die daraus getroffenen Entscheidungen für den Sport die Gesundheit der Kinder und Jugendlichen in Deutschland aufs Spiel setzt und wie Sport, Spiel und Bewegung nun helfen können

**DOI:** 10.1007/s43594-021-00048-3

**Published:** 2021-11-22

**Authors:** Katja Klemm

**Affiliations:** Institut für Sport und Sportwissenschaft, Karlsruher Institut für Technologie, Karlsruhe, Deutschland

## Die Ausgangssituation: Der Alltag von Kindern und Jugendlichen

Im März 2020 änderte sich wohl das Leben fast aller Menschen, für manche schon etwas früher, für manche erst später, aber schlussendlich war es für alle irgendwann irgendwie anders.

**Figure 1:**
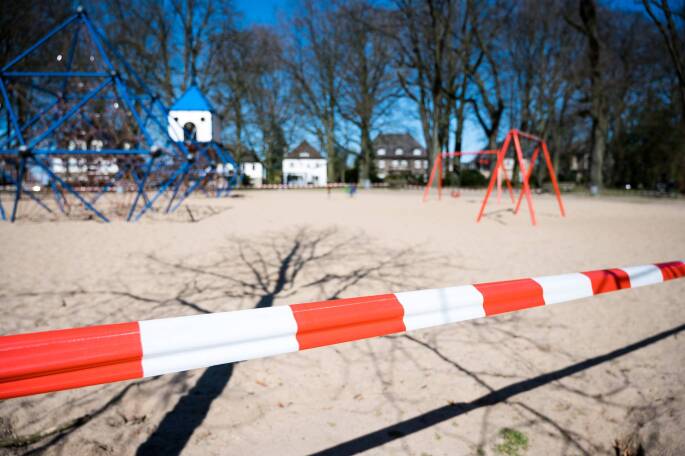

Für die meisten Kinder und Jugendlichen war es wohl erstmal komisch, vielleicht auch kurz ganz cool: alle sind zu Hause, man kann zocken, muss nicht zur Schule gehen, kann sich intensiv mit den Social-Media-Profilen der Freund*innen beschäftigen und einfach etwas entspannen („chillen“, falls man das noch sagt). Das sollte jedem Kind und Jugendlichen nach einigen Wochen sehr bald sauer aufgestoßen sein, wenn erstens absehbar war, dass das Ganze noch etwas dauert und zweitens die negativen Seiten dieses Lockdowns allmählich immer sichtbarer wurden: Freunde und Freundinnen darf man nicht mehr treffen, der Spiel- und Skaterplatz ist gesperrt (Abb. 1), die Eltern sind viel zu Hause, aber haben auch keine Zeit oder sind noch weniger als sonst daheim. Das Training im Verein, der Unterricht in der Musikschule, das Kicken auf dem Bolzplatz, alles fehlt nun doch irgendwie. Und plötzlich interessieren sich die Eltern wieder sehr für den Schulunterricht, sie müssen, denn sie sind nun die Lehrer*innen zu Hause und führen aus, was die echten Lehrer*innen von ihrem Zuhause aus vorgeben.

Doch nicht für alle Kinder und Jugendlichen war diese Situation gleich. Um das nachzuvollziehen, möchte ich zwei Beispiele von fiktiven Kindern in Deutschland aufzeigen^1^, wie ihr Alltag ausgesehenen haben könnte. Im Anschluss möchte ich aufzeigen, was deren Alltag während der Pandemie beeinflusst hat und wie sich das auf das gesunde Aufwachsen der Kinder ausgewirkt hat. Darauf aufbauend soll das folgende Kapitel Möglichkeiten aufzeigen, was Sport, Spiel und Bewegung – in allen Formen – leisten können und zukünftig werden.

Kind A ist neun Jahre alt. Der Junge wohnt mit seiner Mutter und seinem zwei Jahre älteren Bruder in einer mittelgroßen Stadt im Norden Deutschlands. Sie bewohnen eine geräumige Drei-Zimmer-Wohnung am Stadtrand im zweiten Stock, einen Garten oder Balkon haben sie keinen. Er und sein Bruder bewohnen ein Zimmer, um ein Wohnzimmer für alle zu haben. Seine Schule ist fünf Minuten zu Fuß entfernt und er geht wie sein Bruder auf eine Gesamtschule. Nachmittag geht er sehr oft mit seinen Freunden auf den Kickplatz. Wenn es regnet oder sie mal keine Lust haben, gehen sie meist zu einem Freund, um mit der neuesten Spielekonsole zu zocken. Der Junge hat erst vor kurzem das alte Smartphone von seinem Bruder bekommen. Wenn er für die Schule mal mehr braucht, darf er den Laptop seiner Mutter benutzen, der für alle da ist. Seine Mutter ist Büroangestellte in einem nahegelegenen Krankenhaus und arbeitet halbtags. Bei ihrem Vater sind die zwei Jungs jedes zweite Wochenende, er wohnt nicht weit entfernt mit seiner Frau und ihrem Kind in einem EU-Nachbarland und arbeitet in der Baubranche.

Kind B ist 13 Jahre alt. Das Mädchen wohnt mit ihren Eltern in einem kleinen Haus in einem Vorort einer großen deutschen Stadt im Osten Deutschlands. Im Haus hat sie ihr eigenes kleines Zimmer sowie einen angrenzenden kleinen Garten. Die Eltern arbeiten beide in Vollzeit, als Anwältin und Lehrer. Das Mädchen fährt je nach Wetter mit dem Rad oder dem Bus zwei Orte weiter zum Gymnasium. Zwei bis drei Mal die Woche geht sie mittags nach der Schule zu ihren Großeltern mütterlicherseits, die zwischen der Schule und dem zu Hause ebenso in einem Eigenheim wohnen. Zweimal die Woche geht das Mädchen zum örtlichen Leichtathletikverein ins Training. Ansonsten trifft sie sich mit ihren Freundinnen zum Quatschen und Musikhören oder spielt mit ihrer Katze. An den Wochenenden machen sie noch oft Ausflüge mit der Familie, aber ab und zu geht sie auch mit ihren Freundinnen im nächstgrößeren Ort shoppen oder ins Kino.

Und dann kam Corona, die Krankheit, das Virus, der leichte, aber wochenlange Lockdown in Deutschland.

## Was Corona für den Alltag bedeutete

Zuerst war es für beide Kinder sicherlich eine komische, aber noch nicht ganz greifbare und teilweise auch ganz coole Situation: Zur Schule ging es erst einmal nicht mehr, die Eltern sind plötzlich zu Hause und haben mehr Zeit oder im Fall von Kind A sind mehr bei der Arbeit, sodass der Junge mehr sturmfrei mit seinem Bruder hatte. Die Freunde und Freundinnen sind weiter per Social Media zu erreichen und man kann sich weiterhin gut über die Klassenkamerad*innen oder das neueste Spiel austauschen.

Spätestens nach zwei, drei Wochen gibt es aber nicht mehr viel auszutauschen, denn es passiert nicht mehr viel. Der Haupt-Sozialisationsraum für Kinder und Jugendliche mit Gleichaltrigen, die Schule, findet nicht oder auf Sparflamme online statt. Und nun erleben auch die Kinder verschiedene Situationen und sehen sich individuellen Hindernissen gestellt.

Kind A verbringt viel Zeit mit seinem Bruder. Die beiden zocken viel oder schauen fern. Ab und zu nehmen sie ihre Fahrräder und fahren bei Freunden vorbei, nur um sich kurz zu sehen; zusammensitzen oder gemeinsam kicken dürfen sie nicht. Schule haben sie täglich, aber immer nur kurz. Es gibt Aufgaben, die sie sehr einfach finden und mit denen sie nach kurzer Zeit fertig sind. Ganz oft streiten sie sich aber auch, wenn dem kleinen Bruder das Essen vom großen Bruder nicht schmeckt, der große Bruder mal allein das Spiel spielen möchte oder sie unterschiedliche Sendungen im Fernsehen anschauen wollen. Das passiert sehr oft. Abends kommt ihre Mutter nach Hause, sie muss nun mehr arbeiten, da im Krankenhaus sehr viel los ist und immer wieder Kolleg*innen ausfallen, da sie krank sind und es möglicherweise Corona ist. Sie ist sehr streng damit, dass ihre Jungs keine anderen Kinder treffen, da sie in ihrem Job einem hohen Risiko ausgesetzt ist und das Virus keinesfalls verbreiten möchte. Sie bekommt jeden Tag mit, wie viele Menschen an oder mit dem Corona-Virus sterben. Ihren Vater können die Jungs nicht sehen, zuerst war da ein hohes Risiko, jetzt ist die Grenze auch zu. Alle möchten kein Risiko eingehen. Die Mutter ist abends immer sehr müde und geht oft sehr früh ins Bett, die Jungs sind noch wach, haben noch viel Energie und wissen nicht wohin damit. Oft spielen sie noch bis spät am Abend auf ihren Smartphones.

Kind B verbringt viel Zeit mit ihrer Katze im Garten, wenn sie da ist. Ihre Mutter hat das Wohnzimmer zum Homeoffice umgebaut, ihr Vater gibt im Bürozimmer Onlineunterricht. Oftmals muss sie die Kamera bei ihrem eigenen Online-Unterricht ausschalten, da das Internet sonst zu langsam ist. Sie hat täglich zwei bis drei Stunden digitalen Unterricht. Dann erledigt sie ihre Hausaufgaben. Und dann ist ihr langweilig. Ihre Freundinnen darf sie nicht sehen, sie schreiben sich, aber wissen auch schon lange nicht mehr worüber, unter anderem da sie ja die Jungs aus der Klasse auch nicht mehr sehen. Ihre Eltern haben ihr ein kleines Trampolin für den Garten gekauft, das hat kurz Spaß gemacht, aber jetzt möchte sie es auch gern ihren Freundinnen zeigen. Die Großeltern haben es schon gesehen, im Garten treffen sie sich ab und zu mit Abstand. Die Oma backt viel Kuchen und näht Masken, die sie vorbeibringt, aber sie dürfen den Kuchen nicht gemeinsam essen. Mit ihrem Vater zusammen kocht sie oft das Mittagessen, manchmal geht sie auch allein dafür in den Supermarkt. Man soll ja nicht mit der ganzen Familie einkaufen gehen. Abends gibt es oft Diskussionen darüber, was im Fernsehen geschaut wird, normalerweise gibt es diese nicht, da die Familie selten komplett zu Hause ist und im Verein, mit Freund*innen oder bei Verwandten in der Umgebung unterwegs ist.

Wie aus den beiden fiktiven Beispielen herauszulesen ist, sind wohl die offensichtlichsten Einflüsse erstens die Kontaktbeschränkungen zur Peergroup und zweitens die Einschränkungen in der Freizeitgestaltung. Diese beiden Aspekte sind gerade für Kinder und Jugendliche so wichtig, für den Ausgleich zu Schule und Eltern, aber auch für die langfristige Entwicklung. Bei den meisten Kindern und Jugendlichen waren die Eltern wohl so viele Stunden wie lange nicht mehr zu Hause, bei wenigen war es gerade auch das Gegenteil davon, wenn die Eltern in systemrelevanten Berufen arbeiten. Hier waren die Kinder plötzlich sehr auf sich alleine gestellt, da Ersatz-Erziehungsberechtige wie Großeltern, Tanten und Onkel oder auch Eltern von Freund*innen nicht da sein konnten. Für viele Kinder und Jugendliche war es somit ein beschleunigtes Erwachsenwerden, dessen Auswirkungen heute noch nicht abzusehen sind.

**Figure 2:**
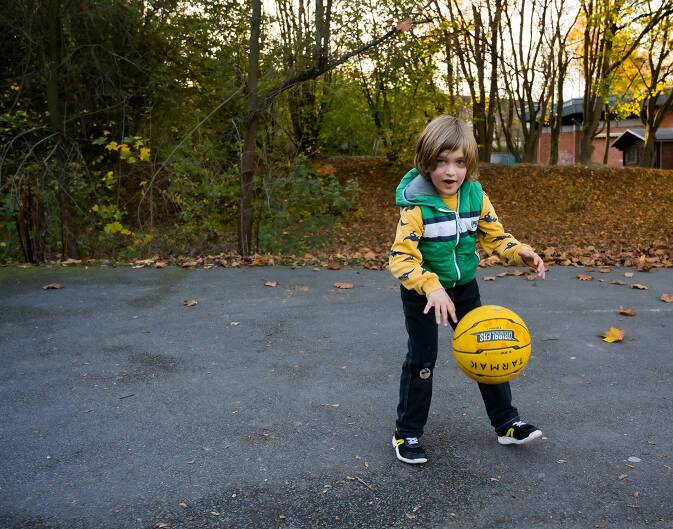

Egal ob das nun im Szenario von Kind A oder B ist: Kinder und Jugendliche hatten keinerlei oder kaum Rückzugsmöglichkeiten zu Hause und auch keine Fluchtmöglichkeiten nach draußen. Sie waren, wie alle anderen auch, gezwungen, sich intensiv mit sich selbst zu beschäftigen (Abb. 2). Das ganze Leben, der Alltag, die Schule, die Freizeit war auf solch einen kleinen Raum begrenzt, der meist noch mit Personen geteilt werden musste, die in bestimmten Phasen nicht unbedingt die ersten Ansprechpersonen von Kindern und Jugendlichen sind. Über soziale Medien und weitere Plattformen der Schule konnte sicherlich gut mit Schulkolleg*innen und Freund*innen Kontakt gehalten werden, aber worüber sollte man sich nach Wochen der Pandemie noch unterhalten?

## Sport, Spiel und Bewegung während Corona

Auch Sport, Spiel und Bewegung mussten viel innerhalb der Wohnung oder des Hauses absolviert werden. Falls das überhaupt möglich war, je nach Platz und Material war vieles sicherlich gar nicht möglich oder nur umständlich, was Hürden bedeutete, die zu überwinden waren oder dann im Weg standen. Bei gutem Wetter wurden sicherlich das Spazierengehen, Joggen und Radfahren ausgiebig wahrgenommen, doch das wichtige Auspowern und an die eigenen Grenzen gehen, das Kinder und Jugendliche ab und zu einfach brauchen, blieb oft auf der Strecke. Ebenso auf der Strecke blieb das gemeinsame Bewegen und Sporttreiben, insbesondere mit der dortigen Peergroup. Egal ob im Verein, Fitnessstudio oder mit den Nachbarkindern auf dem Bolzplatz. Diese Kontakte, die Auseinandersetzung mit Gleichaltrigen im und durch den Sport, fehlte komplett.

„Wieso wird der Sport dann gerade so stark verhindert?“, „Wieso kann ich dann nicht ins Training bei meinem Sportverein/Fitnessstudio/Sportanbieter?“, „Wieso ist dann kein Sportunterricht möglich?“. Diese und viele weitere Fragen kamen auf, als eine große US-Studie im April diesen Jahres (Sallis et al. 2021) von Politiker*innen, Forscher*innen, Sportler*innen und vielen weiteren Personen des (nicht)-öffentlichen Lebens diskutiert, geteilt und zitiert wurde: Inaktivität hängt mit Risiko für schweren Covid-19-Verlauf zusammen! Die Forderung der Autor*innen: Regelmäßige körperliche Aktivität für alle Bevölkerungsgruppen muss dringend in die öffentliche Debatte um die Pandemiekontroll-Regelungen! Bis zu diesem Zeitpunkt war dies die größte Studie zum Zusammenhang von (In‑)Aktivität und der Covid-19-Erkrankung.

Seitdem ist sicherlich viel passiert, und durch das Engagement des Deutschen Olympischen Sportbunds (DOSB), der Deutschen Sportjugend (dsj) und viele weiteren Vereinen und Verbänden konnte eine Öffnungsstrategie auch für den Sport konzipiert werden. Dennoch hat sich gerade durch die Pandemie, die so gerne als Brandbeschleuniger für viele anderen Themen gesehen wird (zum Beispiel mobiles Arbeiten, Digitalisierung in der Bildung), auch in der Sport- und Bewegungslandschaft Deutschland viel getan.

So hat sich besonders während des ersten Lockdowns im April/Mai 2020 die Alltagsbewegung als das letzte noch mögliche Freizeitelement eines sehr reduzierten Freizeitangebots erwiesen, und viele Bevölkerungsgruppen haben dies bei bestem Wetter genutzt. Kinder und Jugendliche hatten einer Untersuchung der MoMo-Studie nach (Schmidt et al. 2021) durchschnittlich mehr Bewegungszeit als vorher. Wobei hier die Einschränkung genannt werden muss, dass es einen großen Unterschied zwischen Bewegung und Sport gibt, der gerade bei Kindern und Jugendlichen eine enorme Rolle spielt. Zudem zeigte sich hier ein Gefälle zwischen Kindern und Jugendlichen in der Stadt und denen, die auf dem Land oder in der Vorstadt leben und einen geschützten größeren Bewegungsradius wie beispielsweise Garten und nahegelegene Wiese haben.

Andererseits haben aber auch viele Vereine mit großem personellem und teilweise sicherlich auch finanziellem Aufwand digitale Angebote wie das Training zu Hause per Videoschalte oder coronakonforme Outdoor-Angebote wie Rallyes geplant, durchgeführt und auch meist für gut befunden. Auch noch in der Freizeit vor einem Bildschirm zu sitzen, war und ist für viele sicherlich nicht optimal, aber sicherlich besser als sich gar nicht zu sehen und die Verbindung zu den Kindern und auch zwischen den Kindern zu verlieren.

## Sport, Spiel und Bewegung und die Gesundheit der Kinder und Jugendlichen

Und damit kommen wir auch schon zu dem, was diese Studie zur Verbindung von Inaktivität und Corona-Krankheitsverlauf mit dem (digitalen) sozialen Miteinander in den Sportvereinen für Kinder und Jugendlichen gemeinsam hat: den Zusammenhang von Gesundheit und sportlicher Aktivität (im Sportverein).

Meine Kollegin Anke Hanssen-Doose konnte in ihrem Forschungsbeitrag „Dauerhaftes Sporttreiben im Sportverein und motorische Entwicklung: Ergebnisse der MoMo-Längsschnittstudie (2003–2017)“ (Hanssen-Doose et al. 2021) anschaulich darstellen, wie der Sportverein als Setting zum Sporttreiben einen sichtbaren Effekt in der motorischen Leistungsfähigkeit der Kinder und Jugendlichen zeigt. Wie sie ebenso darstellt, ist die motorische Leistungsfähigkeit ein wichtiger Gesundheitsmarker im Quer- sowie im Längsschnitt. Ein motorisch fittes Kind ist somit bei Fortführung der körperlichen Aktivität ein gesunder Erwachsener. Diese Verbindung scheint vielen einzuleuchten, ist erwiesen und auch logisch: Wenn ich mich bewege und damit meine Fitness verbessere, bin ich meist gesünder. Dennoch gibt es hier besonders in Bezug auf das Konstrukt der Fitness, also der motorischen Leistungsfähigkeit, noch wenige Untersuchungen. Der Fokus der meisten Studien liegt in der körperlichen Aktivität und deren Wirkung in Bezug auf die Gesundheit (auf verschiedenen Ebenen).

Im Mai jeden Jahres wird in den USA der Mental Health Awareness Month begangen, in dem auf die mentale Gesundheit und deren Zusammenhänge und Ursachen (in positiver und negativer Hinsicht) hingewiesen wird. Nach einer Studie des Universitätsklinikum Hamburg-Eppendorf fühlten sich 71 % der Kinder und Jugendlichen durch die Beschränkungen der Pandemie psychisch belastet (Ravens-Sieberer et al. 2021). Daten der Momo-Studie (Wunsch et al. 2021) bestätigen dies durch Daten der subjektiven Lebensqualität von 8‑ bis 18-jährigen Kindern und Jugendlichen in Deutschland, die während der Pandemie sank.

Die dsj hat im Rahmen des Aktions-Monats ein Positionspapier veröffentlicht, in dem sie besonders hervorhebt, welchen positiven Beitrag Sport, Spiel und Bewegung auf die mentale Gesundheit von Kindern, Jugendlichen und jungen Erwachsenen haben. Sport soll dabei nicht nur als Ausgleich zum stressigen Alltag (auch schon bei Kindern und Jugendlichen!) gesehen werden, sondern als Ventil, als geschützter und als sozialer Unterstützungsraum, in dem sie Verbundenheit und Zugehörigkeit erfahren.

**Figure 3:**
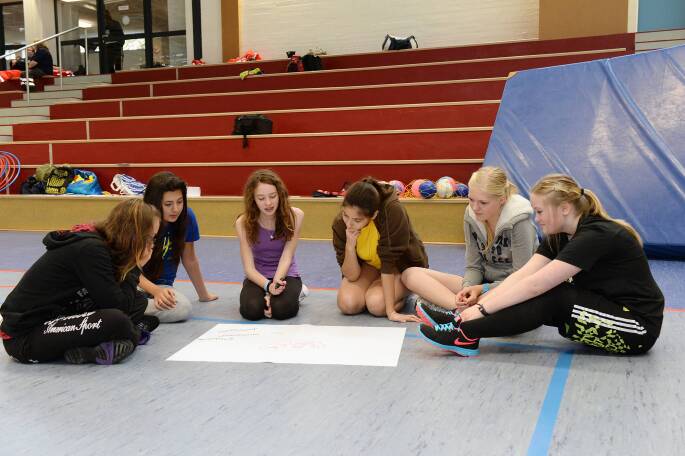

Dieser Raum ist eine weitere wichtige Komponente der Gesundheit: das soziale Umfeld, in dem sich Kinder und Jugendliche ausprobieren können, Werte erlernen und sich als Mensch entwickeln können. Der Sportraum, besonders in Gemeinschaften wie Sportvereinen, lehrt sie das soziale Miteinander auf spielerische Art und Weise zu erleben und auch das eigene Selbst in diesem Gefüge einzuordnen (Abb. 3). Sieg, Niederlage, das gemeinsame Gewinnen und Verlieren, das Engagement von Trainer*in, Eltern und Vereinsverantwortlichen beeinflusst und lehrt Kinder und Jugendliche im Kleinen, was zukünftig auf sie zukommen kann.

Insgesamt gesehen hatte die Pandemiesituation – in all ihren offensichtlichen und weniger offensichtlichen Aspekten – große Auswirkungen auf die Gesundheit von Kindern und Jugendlichen. Egal ob es um die physische Gesundheit, die mentale oder auch soziale Gesundheit geht – diese monatelangen Einschränkungen haben jede*n betroffen. Nun kann, wie oben erwähnt, der Sport in der Gemeinschaft eine große Rolle bei der Aufarbeitung, dem Nachholen und dem Neuaufbau der Gesundheit spielen. Der Sport in der Gemeinschaft, für viele klassischerweise der Sportverein, obwohl dieser sich auch Kritik gegenüber offen zeigen muss. Strukturen sind veraltet, keine jungen Kräfte im Ehrenamt, spießig und altmodisch soll er sein. Teilweise ist das sicherlich auch noch so, aber auch Sportvereine lernen immer mehr, mit der Zeit zu gehen. Sie beschäftigen sich mit Trends, entwickeln Mitgliedssysteme die flexibel sind, entwickeln Mentor*innen-Programme für das Ehrenamt, bauen Calisthenics-Parks in ihre Sportanlagen und schaffen Sport- und Bewegungsangebote, die mit der Zeit gehen. Dennoch sollte nicht das gesamte Konzept des Sportvereins über den Haufen geworfen werden. Es kann ja sein, dass das Konzept eines verbindlichen wöchentlichen Trainings mit der gleichen Peergroup und dem*der gleichen Trainer*in das Beste ist, was den Kindern und Jugendlichen in dieser Situation passieren kann.

Und nicht nur diese Aspekte könnten außerordentlich hilfreich sein. Auch die Inhalte des sportlichen Trainings, das strukturiert aufgebaut ist und den Möglichkeiten der jungen Sportler*innen angepasst ist, sind extrem nützlich für den Aufbau der motorischen Leistungsfähigkeit und damit der physischen Gesundheit. Das freie Spielen und Bewegen ist zwar wichtig und konnte weitgehend fortgeführt werden, jedoch benötigen Kinder und Jugendliche fordernde Reize, die sie sehr gut im strukturierten Training erhalten. So konnte der Fitnessbarometer 2021 (Kinderturnstiftung Baden-Württemberg 2021), eine Analyse der motorischen Leistungsfähigkeit der Kinder und Jugendlichen in Baden-Württemberg, feststellen, dass 2020 im Vergleich zu den Jahren 2012 bis 2019 die Ausdauer und Schnelligkeit abgenommen haben. Die Fähigkeiten Kraft und Beweglichkeit sind angestiegen, was für das funktionierende Online-Training spricht, in dem diese Fähigkeiten gut zu trainieren sind. Die Koordination ist auf ähnlichem Niveau stagniert. Ähnliche und teils noch schwerwiegendere Ergebnisse zeigt eine Untersuchung der Drittklässler in Berlin (Zinner et al. 2021). Hier hat sich lediglich die Ausdauer verbessert. Interessant wird sein, was diese jährliche Erhebung in den nächsten Jahren bezogen auf die Fitness der Jüngsten herausfinden wird.

Sorgen sollte uns hier bereiten, dass die Mitgliederzahlen in Sportvereinen, gerade im Bereich der Kinder, stark gesunken sind. Hier gilt es, die Kinder mit ihren Eltern gemeinsam in den Sportverein (zurück) zu holen. Sicherlich eine tolle Idee, die mehr als nur eine gute Schlagzeile sein sollte, ist das kostenlose Jahr im Sportverein. Nach der Forderung des Deutschen Kinderhilfswerks im Rahmen des Weltspieltags im Mai 2021 wurde diese Idee sehr schnell aufgegriffen, inzwischen hat unter anderem Bayern (Gutschein für Grundschüler*innen) diese Idee umgesetzt. Andere Bundesländer und Gemeinden bieten zumindest kostenfreie Schwimmkurse an, die dieses Defizit schnellstmöglich ausgleichen sollen. Weitere sollten dieser Idee unbedingt folgen und dann auch überlegen, wie das Angebot langfristig aufrechterhalten oder zumindest angepasst am Leben gehalten werden kann.

## Was nun zu tun ist

Ob es nun um die Bewegung im Freien oder den Sport im Sportverein geht, wichtig ist die Erkenntnis der Notwendigkeit und des Potenzials sowie die darauf aufbauende Handlung der verschiedenen verantwortlichen Akteure. Dabei ist es egal, ob es sich nun um Kind A, B oder alle anderen betroffenen Kinder und Jugendlichen handelt. Es geht darum, Aktionen zu starten, Hürden abzubauen und allen Kindern und Jugendlichen den einfachen Zugang zu den Angeboten zu ermöglichen. Das kann nur mit einem Netzwerk aus Politik (Bund, Länder, Kommunen), Wissenschaft, Sportverbänden und -vereinen sowie weiteren gemeinnützigen Organisationen gelingen, die in eine Richtung arbeiten zum Wohle der Gesundheit von Kindern und Jugendlichen auf allen Ebenen.

Die Pandemie hat nach meiner Ansicht dazu geführt, dass vieles offen gelegt wurde, vielen wurde klar, was Priorität hat, und was eben nicht. Das allein auf die Politik zu schieben macht es sicherlich leicht, ist aber nicht nachhaltig. Was nun wirken und zählen kann, ist ein gemeinsames Ziehen an einem Strang, um Kindern, Jugendlichen und jungen Erwachsenen und damit den Erwachsenen von morgen und übermorgen ein gesundes Leben zu ermöglichen.

## Footnotes

1 Jede Ähnlichkeit zu realen Kindern und Jugendlichen ist zufällig.
